# Early Lens Ablation Causes Dramatic Long-Term Effects on the Shape of Bones in the Craniofacial Skeleton of *Astyanax mexicanus*

**DOI:** 10.1371/journal.pone.0050308

**Published:** 2012-11-30

**Authors:** Megan Dufton, Brian K. Hall, Tamara A. Franz-Odendaal

**Affiliations:** 1 Department of Biology, Dalhousie University, Halifax Nova Scotia, Canada; 2 Department of Biology, Mount Saint Vincent University, Halifax Nova Scotia, Canada; University of Sheffield, United Kingdom

## Abstract

The Mexican tetra, *Astyanax mexicanus*, exists as two morphs of a single species, a sighted surface morph and a blind cavefish. In addition to eye regression, cavefish have an increased number of taste buds, maxillary teeth and have an altered craniofacial skeleton compared to the sighted morph. We investigated the effect the lens has on the development of the surrounding skeleton, by ablating the lens at different time points during ontogeny. This unique long-term study sheds light on how early embryonic manipulations on the eye can affect the shape of the adult skull more than a year later, and the developmental window during which time these effects occur. The effects of lens ablation were analyzed by whole-mount bone staining, immunohistochemisty and landmark based morphometric analyzes. Our results indicate that lens ablation has the greatest impact on the skeleton when it is ablated at one day post fertilisation (dpf) compared to at four dpf. Morphometric analyzes indicate that there is a statistically significant difference in the shape of the supraorbital bone and suborbital bones four through six. These bones expand into the eye orbit exhibiting plasticity in their shape. Interestingly, the number of caudal teeth on the lower jaw is also affected by lens ablation. In contrast, the shape of the calvariae, the length of the mandible, and the number of mandibular taste buds are unaltered by lens removal. We demonstrate the plasticity of some craniofacial elements and the stability of others in the skull. Furthermore, this study highlights interactions present between sensory systems during early development and sheds light on the cavefish phenotype.

## Introduction

The eye is an island of soft tissue surrounded by the neural crest derived tissues of the craniofacial skeleton. Studies investigating interactions between the soft eye tissues and the harder skull tissues have been rare until recently. Here, we explore this relationship and demonstrate that some bones are plastic, while others are resistant to manipulation. To do this we use a fish species which is extremely well suited for studying the influence of the eye on craniofacial development, namely the Mexican tetra.

The Mexican tetra, *Astyanax mexicanus*, currently thought to be a single species, which exists as two morphs, a surface morph and a cavefish morph [1], [2], [3]. The sighted, surface morph is found in streams and rivers throughout Mexico [4]. The approximately 29 different blind cavefish morphs are thought to have originated from the surface morph in the last few million years [5]. These cavefish populations are found in a small region of limestone caves located in north-eastern Mexico [6]. The most striking and most highly investigated feature of the cavefish is their lack of functional eyes in adulthood. Eye development begins in the same manner in both the surface and cavefish morphs; however degeneration of the early cavefish eye primordia begins as a result of apoptosis in the lens [7], [8]. In addition to eye degeneration, cavefish have other regressive changes including loss of pigmentation, reduction in the size of the optic tectum, and loss of schooling behaviour. Less commonly studied are the constructive changes, which include changes in body position while feeding, increased jaw size, increased number of taste buds, increased size and number of neuromasts, larger fat stores and increased number of maxillary teeth [9], [10].

We are investigating the interactions that occur between the developing eye and the surrounding ocular skeleton. Eyed and eyeless forms of the Mexican tetra are an excellent model system for such studies. The skeleton surrounding the eye of the surface morph consists of one supraorbital bone and six suborbital bones, collectively known as circumorbital bones. Cavefish have a unique skeletal morphology surrounding the eye differing from surface fish in number of elements, size, and position of suborbital bones [11]. These bones are intramembranous bones that form directly from mesenchymal condensations. Circumorbital bones are late-forming bones which begin to ossify at approximately 22 mm body length, which is around the age when scales develop [11]. In addition, the surface fish (as in many other teleosts) have two endochondrally formed scleral ossicles in the eye [12], [13], while cavefish have a single cartilage element in the sclera [11], [13].

In a pioneering study conducted 10 years ago, Yamamoto and Jeffrey (2000) demonstrated that transplantation of a cavefish lens into a surface fish eye at 24 hours post fertilization (hpf) induces eye regression in the surface morph, mimicking cavefish eye regression [7]. When eye regression was induced in this manner some craniofacial bones were affected; that is some bones appear to be dependent on the presence of the developing eye [11]. Dependent elements include: the distance between the nasal bone, antorbital bones and olfactory pits, an ossified sclera (i.e. scleral ossicles), and the shape of both suborbital 3 and the supraorbital bone. Elements not altered by the presence of the eye (i.e. independent) include the maxillary teeth, the number of elements making up suborbital 3,the positions of suborbitals 4 through 6, and the shape of the opercular bone [11]. In these studies, however, a subsequent lens transplantation was conducted and therefore the effect of lens removal alone was not clearly established.

Other aspects of head development are different in cavefish, namely tooth number and taste buds. Cavefish have more maxillary teeth and many more taste buds. During normal tooth development, small unicuspid teeth develop in the centre of the jaws (maxilla, premaxilla and mandible) and are later replaced by large multicuspid teeth. As adults, Mexican tetras have both large multicuspid teeth in the center of the lower jaw as well as smaller teeth on the caudal portion of the jaw. These small caudal teeth are uni- and multi-cuspid, and vary in number during adulthood [14]. With respect to taste buds, our lab recently documented the number of taste buds present in an ontogenetic series of cavefish and surface fish [15], [16]. Cavefish were found to have a significantly larger number of taste buds compared to surface fish by 22 dpf and by adulthood this translates into a five to seven fold difference [15], [16].

These regressive and constructive changes in the cavefish have intrigued scientists for many years. It was hypothesized that the sensory organs of the Mexican tetra can be regarded as individual, yet linked modules [17]. Some modules are expanded in the cavefish, while others are regressed. Modules exist as networks of gene expression, cell types and developmental processes; natural selection may act on modules at any of these levels [17]. In 2009, Yamamoto and colleagues investigated this further through over expression of *shh* in surface fish and inhibition of *shh* in cavefish [18]. Following ectopic *shh* expression the authors report an increase in taste bud number and mandible size. In addition, the authors concluded that the midline expression of the gene *sonic hedgehog (shh)* has pleiotropic effects during early development and that a sensory module trade-off may exist between the loss of eyes (eye module), increase in taste buds (gustatory module) and increase in lateral line neuromasts [18]. Further investigation regarding the role of *shh* in cavefish eye reduction using quantitative trait loci analysis indicates that mutations in this gene are unlikely to be directly responsible for the loss of functional eyes [19].

In the current study, we investigate the links between the development of the eye and the surrounding craniofacial skeleton. To do this we ablated the lens from surface tetra embryos and analyzed the adult skull and associated structures. We conducted lens ablation in surface fish at multiple time points and have examined the effects in older adults than previously examined by Yamamoto and colleagues [11]. In addition, we carefully examined the shape changes in the circumorbital (the supraorbital and suborbitals) bones surrounding the eye. Our results indicate that many (but not all) of the circumorbital bones are affected by lens removal; highlighting the plasticity of some skull bones. Our results also show that the earlier the lens is removed the greater the impact on the surrounding skeleton. We determined which parts of the teleost skull are plastic and variable as a result of physical manipulation of the eye and which are stable. Surprisingly, our results also indicate that while mandibular taste bud number is unaffected, the number of small teeth in the caudal (posterior) region of the mandible is affected by the absence of the developing eye. This type of long term study is unique and provides valuable insight into our understanding of how processes during early stages of eye development can affect the adult skull.

## Materials and Methods

### Ethics statement

All protocols follow the Canadian Council on Animal Care guidelines and were approved by the SMU-MSVU Animal Care Committee (protocol numbers 10–21, 11–16, 12–21).

### Biological material, Mexican tetra, *Astyanax mexicanus*

Surface Mexican tetra and Tinaja cavefish adults were obtained from R. Borowsky (New York University, New York City, USA). Adults were maintained at 21°C on a 12 hour light, 12 hour dark cycle. To induce spawning, tank temperature was increased to 26°C and two males were added to a tank containing one female. Eggs were collected the next day. Fish are housed at Mount Saint Vincent University, Halifax Nova Scotia, Canada. Anesthesia was used with all surgeries.

### Lens ablation

Lens removal was conducted unilaterally in surface fish at 24 hpf (n = 6), two dpf (n = 8), three dpf (n = 5), or at 4 dpf (n = 5) using tungsten needles and following the basic procedure outlined by Yamamoto and Jeffery (2002) [20]. Minor alterations included incubating the specimens in 0.01% Ms222 (Ethyl-3-aminobenzoate methanesulfonic acid salt, Sigma E10521) in calcium free zebrafish Ringer's solution for two minutes to anaesthetize the fish. After surgery, specimens were rinsed in zebrafish Ringer's solution three times, released from the agar mounting medium and returned to the fish facility. The non-surgically manipulated eye serves as a control. All experiments were conducted in Nova Scotia, Canada from 2008 until 2011.

### Whole mount bone stain

Adult surface fish, Tinaja cavefish and surgery specimens were anaesthetized using 0.1% MS222, and then fixed in 10% Neutral Buffered Formalin (Fisher, 23-245-685). Alizarin red (Sigma A5533) was used to bone stain the skeleton of adults of a minimum standard length of 3.5 cm, when the ocular skeleton is known to be fully developed, typically between 9 months and 12 months of age [12]. An artificial space is present between the surgery and control sides of the dorsal skull as an incision along the sagittal suture was made during the staining process to allow for better stain penetration. Briefly, fish were bleached overnight in 3% Hydrogen peroxide in 1% Potassium hydroxide (Sigma 1767) solution. The following day, fish were rinsed in water, and then soaked in saturated Sodium tetraborate (Sigma B9876) for 8 hours. Fish were stained overnight in Alizarin stain (1 mg/ml Alizarin in 1% Potassium hydroxide). Finally, specimens were rinsed in 1% Potassium hydroxide then cleared in 1% Trypsin (Fisher Scientific cat, 9002-07-7) and 2% Sodium tetraborate in distilled water for three nights. The specimens were processed through an ascending series of glycerol in 1% Potassium hydroxide solution then transferred to a storage solution of 100% glycerol.

### Morphometric analysis

**Figure 1:**
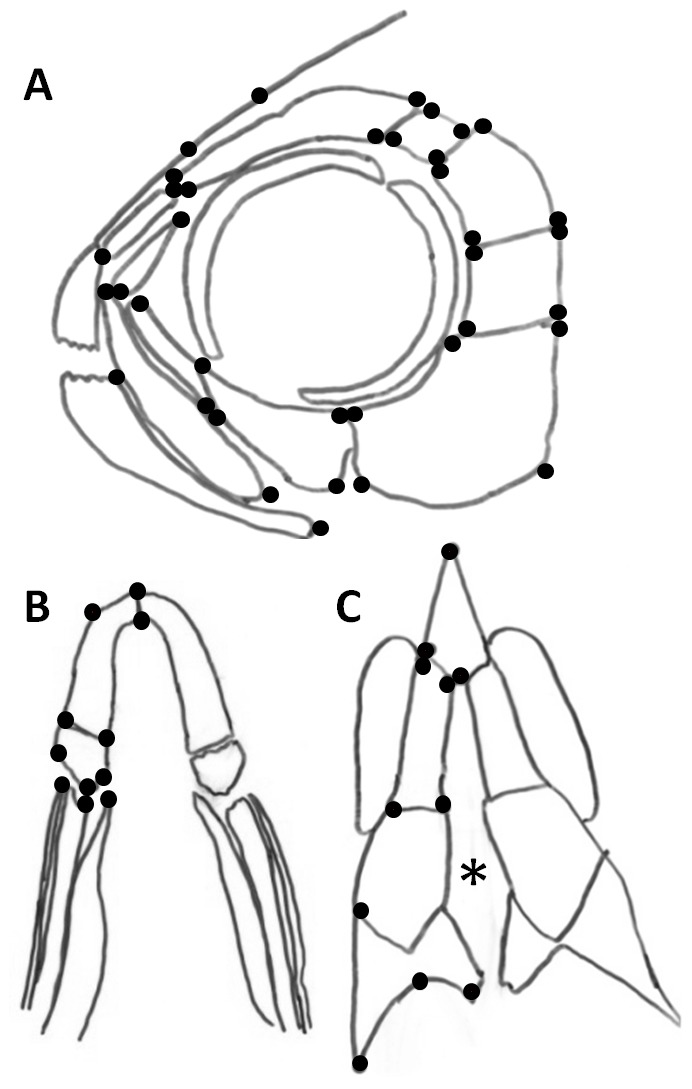
Schematic showing morphometric landmark locations on adult surgery skulls. (A) 42 lateral view landmarks locations; (B) 11 ventral jaw landmark locations; (C) 11 dorsal skull landmark locations. The asterisk indicates the incision in along interfrontal suture.

To compare the control eye to the surgery eye images of the lateral, dorsal and ventral view were captured using a Nikon SMZ1000 microscope and NIS elements software package. Independent analyzes were conducted for each of the four time points of lens removal. Forty-two two-dimensional (x,y) landmarks were applied to the lateral view images of the head (Figure 1, Table S1). Eleven landmarks were applied to each of the dorsal skull and ventral jaw views (Figure 1, Tables S2 and S3). Landmarks were applied using tspDIG2 software (F. James Rohlf, http://life.bio.sunysb.edu/morph/) and analysis was conducted using the IMP series of software (H. David Sheets, http://www3.canisius.edu/~sheets/morphsoft.html). The IMP software Coordgen was used to calculate Procrustes distance and the program TwoGroup was used to calculate the average Procrustes distances for each of the control and surgery eye groups. The statistical difference in shape between the surgery and the control lateral, dorsal and ventral views based on average Procrustes distances was analyzed using Goodall's F-test and F test, Procrustes. This tests the overall shape difference between two groups while taking into account variance within each group. In addition, the lateral view of the surgery side of the skull was compared to control adult tetras that had not undergone surgery on either side of the head.

Vector analyses were conducted using the program tpsSuper written by F. James Rohlf (State University of New York). TpsSuper was used to create a consensus configuration of the landmarks for each of the four control groups and each of the surgery groups. The consensus of each surgery group was compared to its control group using tpsSplin (F. James Rohlf). Vector outputs depict the direction and magnitude of each surgery landmark is different from its corresponding average control. Thin-plate splines were calculated by comparing the average control to the average surgery in the tpsSplin program. Thin-plate splines allow visualization of shape changes in the landmarked locations based on deformation of a grid.

### Immunohistochemistry for visualization of taste buds

Specimens in which the lens was removed at either 1 or 3 dpf were prepared for immunohistochemisty at 21–22 dpf. After fixation in 4% Paraformaldehyde (Sigma P6148) in 0.01 M Phosphate buffered saline, pH 7.4, the immunohistochemistry procedure outlined in Varatharasan et al, (2009) was used to visualize receptor and basal cells of taste buds, using anti-calretinin and anti-serotonin respectively. Taste buds are visualized as one basal cell surrounded by multiple receptor cells. Isolated basal cells were identified but were not included in taste bud counts. Permeabilization was increased to four nights in 4% Trition-x 100 (BDH Chemicals R06433) in 0.01 M Phosphate buffered saline. Two primary antibodies were used, a rabbit monoclonal anti-serotonin (Sigma, s5545) used at a concentration of 1∶10000 to visualize basal cells and a mouse monoclonal anti-calretinin (Abcam, ab90632) at a concentration of 1∶175 to visualize receptor cells. The primary antibodies were incubated at 4°C for four nights, while secondary antibodies were incubated for 48 hours. The two secondary antibodies used were a goat anti-rabbit Alexaflour 488 (Invitrogen, A11034) at a concentration of 1∶500 and bovine anti-mouse Texas red (Santa Cruz Biotechnology, sc-2788) at a concentration of 1∶400. After staining, the jaws were removed and flat mounted. Taste buds were counted on both the right and the left side of the mandible. A one-tailed paired t-test was performed on the numbers of taste buds to determine the significance of these results. Statistical analyses were conducted in Minitab version 16.

### Counting of teeth and jaw measurements

The number of small caudal teeth and large central multicuspid teeth on each half of the lower jaw was documented for surgery specimens at 1, 2, 3, and 4 dpf, after staining with alizarin red. Small caudal teeth consisted of uni- and multi-cuspid teeth and were less than 50% smaller than the large central multicuspid teeth. Jaw measurements were conducted from the retroarticular joint of the dentary to the anterior most point of the dentary. Statistical analyses (one-tailed paired t-test) were conducted on measurements from both the surgery and control side of the lower jaw; statistical analyses were conducted in Minitab version 16.

## Results

### Early lens ablation most dramatically affects the size and shape of the temporal group of suborbital bones

**Figure 2:**
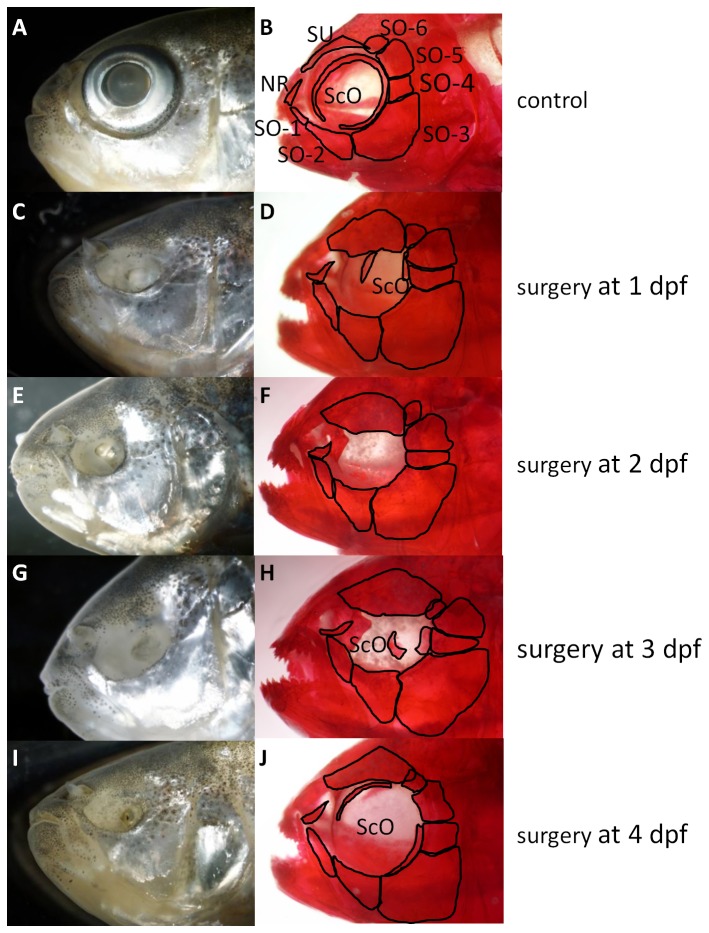
Adult skull of surface morph Mexican tetras. A and B controls; C–J are surgery fish. First column is unstained while fish in the second column are stained with Alizarin red. Surgery eyes have smaller orbits and some bones expand into the orbit (see text for details). NR nasal region, ScO scleral ossicles, SO suborbital bone 1 to 6, SU supraorbital bones.

Lens ablation in the surface Mexican tetra results in eye regression (i.e. a small eye sunken into the head) in approximately 50% of juveniles, regardless of the age of the embryo when surgery is performed (n = 51). The diameter of the unstained regressing surgery eye was measured in live specimens and was compared to the diameter of the normal non-surgery eye. The surgery eye is approximately 30% smaller than the control eye by 30 days post surgery. As the skull grows in size, it appears to outgrow the regressing eye, resulting in the regressed eye becoming sunken into the head and covered with fat and skin (Figure 2C–J).

**Figure 3:**
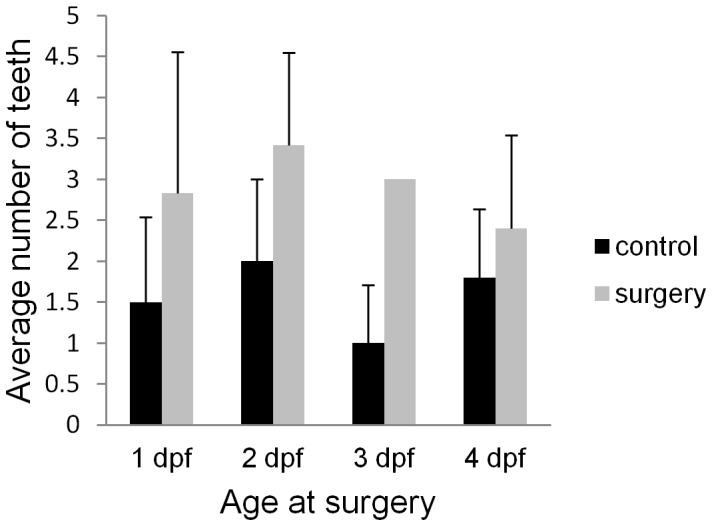
The effect of lens ablation on mandibular teeth. The average number of unicuspid teeth on the surgery versus control side of the head is shown by the bar graph at surgery time point (1, 2, 3, 4 dpf) (n = 6, 7, 5, and 5 respectively).

Several bones surrounding the surgery eye are affected by lens ablation; these effects are less dramatic the later surgery is performed (Table 1, Figures 2 and 3). The supraorbital bone and suborbital bones four through six (the temporal group of circumorbitals) are the most dramatically altered in size and shape following lens ablation (Table 1). In general, these bones are expanded into the orbit. Orbit size decreases as a result of expansion of the bones surrounding the eye. The number of mandibular teeth is also altered as a result of lens ablation. Morphometric and statistical analyses of these differences are discussed below.

**Table 1:** The effect of lens ablation performed at 1, 2, 3 and 4 dpf on the bones surrounding the orbit.

Region	1 dpf	2 dpf	3 dpf	4 dpf
**Anterior scleral ossicle**	absent in 3/6 individuals; highly reduced when present	absent in 5/8 individuals; reduced when present	absent in 3/5 individuals; normal when present	absent in 1/5 individuals; normal when present
**Posterior scleral ossicle**	Present in 1/8 individuals, but reduced in all	absent in 2/8 individuals; large when present	Present in 0/5 individuals and normal	Present in 0/5 individuals and normal
**Supraorbital bone**	largely expanded in all directions	largely expanded in all directions	expanded in all directions	expanded in all directions
**Suborbital 1**	slightly expanded posteriorly and into the orbit	slightly expanded into the orbit and shifted posteriorly	expanded into the orbit and shifted anteriorly	expanded into the orbit and shifted anteriorly
**Suborbital 2**	slightly expanded into the orbit and shifted posteriorly	slightly expanded into the orbit and shifted posteriorly	expanded into the orbit and shifted anteriorly	expanded into the orbit and shifted anteriorly
**Suborbital 3**	expanded into the orbit and shifted posteriorly	expanded into the orbit and shifted posteriorly	expanded into the orbit and shifted anteriorly	expanded into the orbit and shifted anteriorly
**Suborbital 4**	narrower and elongated; expanded into the orbit	narrower and elongated; expanded into the orbit	elongated and shifted into the orbit	elongated and shifted into the orbit
**Suborbital 5**	expanded into the orbit, wedge shaped, shifted ventrally	expanded into the orbit, wedge shaped, shifted ventrally	expanded into the orbit, wedge shaped, shifted ventrally	expanded into the orbit and into a wedge shape, shifted ventrally
**Suborbital 6**	displaced by the supraorbital	displaced by the supraorbital	displaced by the supraorbital	less displaced
**Upper jaw**	unaffected	unaffected	unaffected	unaffected
**Lower jaw**	unaffected	unaffected	unaffected	unaffected
**Nasal region bones (antorbital bone, nasal bone, frontal bone, maxilla)**	slight shift posteriorly	slight shift posteriorly	shifted dorsally	shifted dorsally

The number of individuals affected over total number of individuals analysed are given.

The supraorbital bone is the bone that is most influenced by lens removal (Figure 2, Table 1). Lens removal causes the supraorbital bone to expand in all directions, demonstrating its plasticity. The element is expanded from a thin concave bone above the eye to a large flat plate with a flat ventral edge expanded into the orbit. When surgery is performed at 1 dpf, the supraorbital bone also expands into the normal position of suborbital six (SO6), overlapping this bone. When lens removal is performed later (2 to 4 dpf), however, the effects on the supraorbital bone are less dramatic, with very little expansion in any of the suborbital bones (Figure 2).

Suborbital six (SO6) is displaced by the supraorbital bone and by suborbital five after 1 and 2 dpf surgeries. On the control side SO6 is rectangular in shape and slightly overlaps the supraorbital bone, while on the surgery side of the head, SO6 has expanded into a triangular shape and is wedged adjacent to suborbital five and largely overlaps the supraorbital bone (Figure 2).

Suborbital 5 (SO5) is largely expanded into the orbit after surgery at 1 or 2 dpf. This normally rectangular shaped bone expands into a larger triangular shaped bone with the longest axis extending into the orbit (Figure 2). Suborbital four (SO4) changes from a large square bone to a long thin rectangular bone, also extending into the orbit (Figure 2). Similar to the supraorbital bone and SO6 the effects of lens removal on SO4 and SO5 are most dramatic when performed at 1 and 2 dpf.

In contrast to the above however, suborbital three (SO3), the largest bone in the circumorbital complex is minimally affected by lens ablation, indicating that the shape of SO3 is more stable. The dorsal edge of this bone has slightly expanded into the orbit, but otherwise remains relatively the same shape and size as the corresponding bone on the non-surgery side of the head (Figure 2). Suborbital bones one and two appear expanded into the orbit and are lengthened. The nasal region of the skull (anterior end of SO1, antorbital bone, nasal bone, frontal bone, and the maxilla) remains largely unaltered after embryonic lens removal, with only a minor expansion of each bone into the orbit (not shown).

### Later lens ablation affects small caudal mandibular teeth

Previous investigation determined that cavefish have an increased number of maxillary teeth when compared with the surface fish [11]. Our brief analysis of maxillary teeth numbers after lens removal agree with the findings of the Yamamoto (2003) study indicating that lens removal does not influence the number of maxillary teeth. Furthermore, we examined the number of teeth located on the lower jaw, which was not examined in the previous study. We determined that when surgery was performed at 1 dpf the control side of the lower jaw has on average 1.5±1.04 small teeth, while the surgery side has on average 2.8±1.72 small teeth (Figure 3). This slight increase is not statistically significant (one-tailed paired t-test, p>0.05, n = 6). Interestingly, one-tailed paired t-tests show that lens removal conducted at 2, 3 or 4 dpf has a significant impact on the number of teeth (p<0.05 for each time of surgery, n = 7, n = 5 and n = 5 respectively) with more teeth on the surgery side. When lens removal was conducted at 2 dpf the control side has on average 2.0±1.0 teeth, while the surgery side has 3.42±1.13, after surgery at 3 dpf the control side has 1±0.70 teeth, while the surgery side has 3±0 teeth. Finally after surgery at 4 dpf the control side of the jaw has 1.8±0.833 small teeth, while the surgery side had 2.4±1.14. These results indicate that lens ablation performed later in development has a greater impact on tooth number than when surgery is performed earlier (i.e. at 1 dpf). A constant number of large multicuspid teeth is present in all samples (8 teeth per jaw) and no differences are detected on the surgery versus the control sides.

In addition, no gross morphological differences were present in the length or width of the jaw after lens ablation. Despite this, we measured jaw length and determined that lens removal does not significantly affect the length of the dentary on each side of the head (one-tailed paired t-test; p>0.05, n = 4, n = 3, n = 3, n = 2 respectively).

### Morphometric analyzes shows significant changes in the shape of the circumorbital bones

In order to statistically compare the shape differences in the bones on the control side of the head to the surgery side (described above), morphometric analyses was conducted by applying 42 landmarks on the lateral views of both the control and surgery sides of the skull (Figure 1). Procrustes superimposition was used to align corresponding landmarks in order to analyze these changes in shape. Goodall's F-test indicate that surgery performed at 1, 2, 3 or 4 dpf is significantly different from the control (p<0.0001, n = 6, n = 8, n = 5 and n = 6 respectively). These results agree with the findings from the resampling test F test, Procrustes (p<0.01, F-score: 7.18, p<0.01, F-score: 9.36, p<0.01, F-score: 6.99, and p<0.05, F-score: 3.08, respectively). These drastic changes observed in the gross morphology cannot be accounted for by any minor natural right/left asymmetry that may be present in the skull.

In addition, the surgery side of the head was compared to control fish that had not received surgery. Results from Goodall's F-test indicate that the surgery side is significantly different in shape from control fish (p<0.0001) for each surgery time point. The morphometric program Twogroup indicates that the surgery side (of a 1 dpf surgery) is more similar to the control side of the head than it is to control fish without surgery (range = −0.0727 to 0.0395), the distance between the surgery side and the control side has a p value of 0.1688, while the distance between surgery side and control fish has a p value of 0.1855.

**Figure 4:**
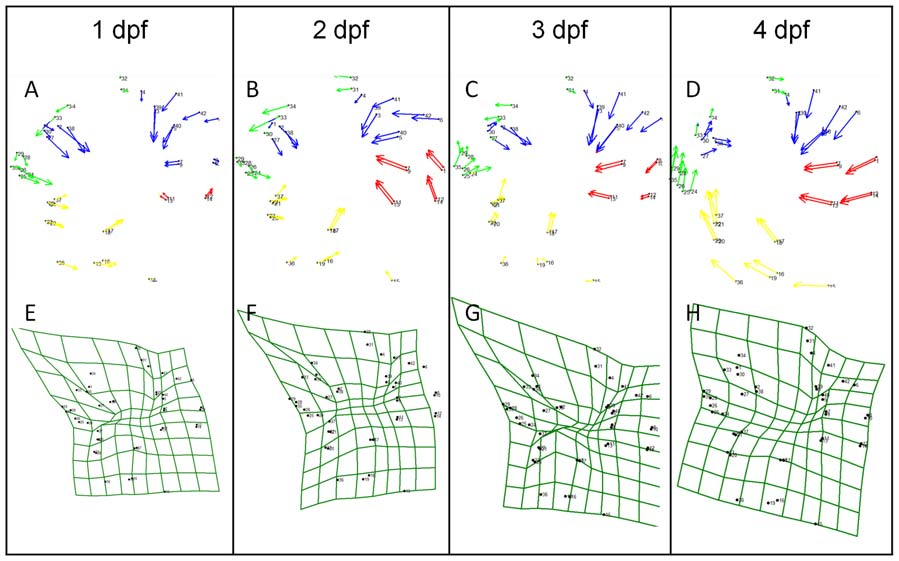
Vector analyzes, and thin plates spline morphometrics of lateral view surgery adults. (A)–(D) vector analyzes comparing the control side of the head to the surgery side. Vectors are grouped based on their similar response to surgery. Group one consists of landmarks 1–10 and 39–42 (blue), group two has landmarks 11–14 (red), group three has landmarks 15–23 and 36–37 (yellow) and group four has landmarks 24–30 and 33–36 (green). (E–H) are thin plate splines of surgery conducted at 1 to 4 dpf.

Vector analyses allow visualization of how and to what degree landmarks shift when comparing control versus surgery sides of the specimens. Vectors were created using a consensus or average for each surgery and each control group. We grouped landmarks into clusters based on similar responses to lens removal. These groups are as follows; landmarks 1–10 and 39–42 (blue), landmarks 11–14 (red), landmarks 15–23 and 36–37 (yellow) and landmarks 24–30 and 33–36 (green) (Figure 4, Table S4).

The group one landmarks (1–10 and 39–42, blue in Figure 4) correspond to the bones directly above the orbit (i.e. the supraorbital bone and SO4–6 to the right of the orbit). These bones are shifted ventrally toward the center of the orbit as indicated by the vector directions. The more ventrally located landmarks in this group (landmarks 1–3, 5, 7, 9, 30, 39, 40) have vectors of larger magnitude than the dorsal landmarks (landmarks 6, 8, 10, 30, 41, 42) indicating that these bones have expanded and shifted. At 1 dpf, the major change is in the size of the bones; the dorsal to ventral axis is expanded as a result of surgery. The directional effect of lens removal on group 1 does not appear to vary with age at the time of surgery. However, the length of the vectors in this group is different in earlier surgery fish and similar in later surgery fish (Figure 4). These vectors also have a greater magnitude the earlier surgery is performed, with the greatest effect at 1 dpf and the least at 4 dpf. The remaining three groups respond differently to lens removal based on the age at which the lens was removed.

Groups two, three and four are less affected by lens ablation and thus will be described more briefly. Generally these groups tend to respond to surgery at 1 and 2 dpf in one manner and respond to surgery at 3 and 4 dpf in a different manner. When lens removal is conducted at 1 or 2 dpf, these groups respond in the following ways. Group two (Figure 4, red) vectors generally show a movement into the orbit, with the anterior landmarks having greater magnitude indicating that SO4 (landmarks 9–12) has elongated into the orbit. Group three (Figure 4, yellow) vectors point in the posterior direction indicating that SO2–3, and the posterior portion of SO1 has shifted and expanded posteriorly. The vectors have small magnitudes indicating that there are small changes to these bones. Group four (Figure 4, green) vectors have a small magnitude and generally point in the posterior and downward direction. This indicates that lens removal has less of an impact on group 4. Group 4 vectors indicate that the bones present to the anterior of the orbit (i.e. in the nasal region) shift slightly in the posterior direction and with a slight expansion into the orbit.

Landmark groups two, three, and four respond differently to lens removal conducted at 3 and 4 dpf than they do to surgery at 1 to 2 dpf. When lens removal was performed at 3 or 4 dpf, group two landmarks respond by shifting in the anterior and slightly ventral direction, indicating that SO4 has shifted into the orbit. After surgery at 3 dpf, the posterior landmarks in group 2 have moved less than the anterior landmarks indicating that SO4 has elongated. Group three landmark vectors point dorsally and slightly anteriorly after surgery at 3 and 4 dpf. The greater magnitude of the dorsal landmarks indicate that the SO1–3 have expanded into the orbit and have shifted anteriorly. Surprisingly, group three is more highly influenced by later surgery then earlier surgery, opposite to the effect that was observed in group one. Group four landmarks are shifted in the dorsal direction as a result of lens removal performed at 3 or 4 dpf.

Overall our results indicate that group 1 landmarks (dorsal and posterior to the eye) are affected to the greatest extent with the most dramatic effects after lens ablation at 1 or 2 dpf. The effects of lens removal at 3 and 4 dpf were milder, with the greatest impact to group three and four. These results support our gross morphology findings described previously. In addition, using this method has allowed us to further understand in what direction the bones have expanded and in what direction they have shifted as a result of lens removal compared to the control side of the head.

### Morphometric analyzes of the effects of lens ablation on the calvariae and mandible

Morphometric analyses were also conducted on the calvariae and the lower jaw (ventral view) to determine if any effects of lens ablation could be observed in these regions. Thin plate splines of specimens in which the lens was removed at 1 dpf show that the calvariae and mandible are not influenced by lens removal (calvariae: Goodall's F-test, p>0.05 n = 5, n = 4, n = 3, and n = 2 for surgery at 1, 2, 3 or 4 dpf; mandible: Goodall's F-test, p>0.05, n = 4 and n = 3 for 1 day and 3 day respectively). Surprisingly surgery performed at 2 and 4 dpf shows a significant difference in shape of the lower jaw on the surgery side compared to the control (Goodall's F-test, p<0.01, p<0.03, n = 4 and n = 3 respectively). Changes in shape were present in the posterior most portion of the dentary, the anguloarticular and the retroarticular. There appears to be a slight expansion in the width of the most posterior structures, however these expansions were very minor. No changes were observed in the anterior portion of the jaw. Similarly, no changes in jaw length were detected as reported above.

### Early lens ablation results in scleral ossicles that are reduced or absent

Normally, sighted Mexican tetras have a large anterior and a large posterior scleral ossicle; these ossicles fuse into a solid bone ring during late adulthood [12]. Lens ablation and subsequent eye regression causes a wide variety of changes in the ossicles (Table 1), ranging from large normal appearing elongated ossicles to small abnormal disk-like or completely absent elements (Figure 2).

When manual lens removal was performed at 1 dpf the majority of individuals have a small posterior ossicle. Fifty percent of surgery specimens with a regressed eye have an anterior ossicle, however, when present it is highly reduced in size (Figure 2). In contrast, lens removal at 2 dpf results in the majority of fish having a large posterior ossicle arching around the eye, while only half of the specimens have anterior ossicles. When anterior ossicles are present they tend to be larger than those present in 1 dpf surgery specimens. Lens ablation at 3 dpf resulted in all individuals developing posterior ossicles, the majority of which were large and normal in shape; 60% of these individuals also had reduced anterior ossicles. Lens ablation performed at 4 dpf results in 100% of the specimens with a large posterior and a large anterior ossicle.

In conclusion, our results show that the anterior ossicle is more affected by lens ablation conducted at 1–3 dpf, while the posterior ossicle remains relatively unaltered. Lens ablation at 4 dpf results in minor changes in scleral ossicle development (Table 1).

### Lens ablation does not affect mandibular taste bud number or pigmentation

**Figure 5:**
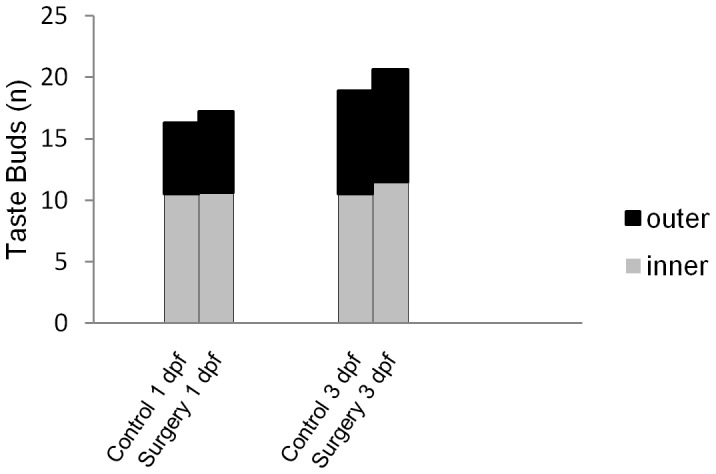
The affects of lens removal on taste bud number. The average number of taste buds on the surgery side of the lower jaw compared to the control side of the lower jaw. Both the inner and outer rows of taste buds were counted. Surgery was performed at 1 and at 3 dpf (n = 19, and n = 8 respectively).

**Figure 6:**
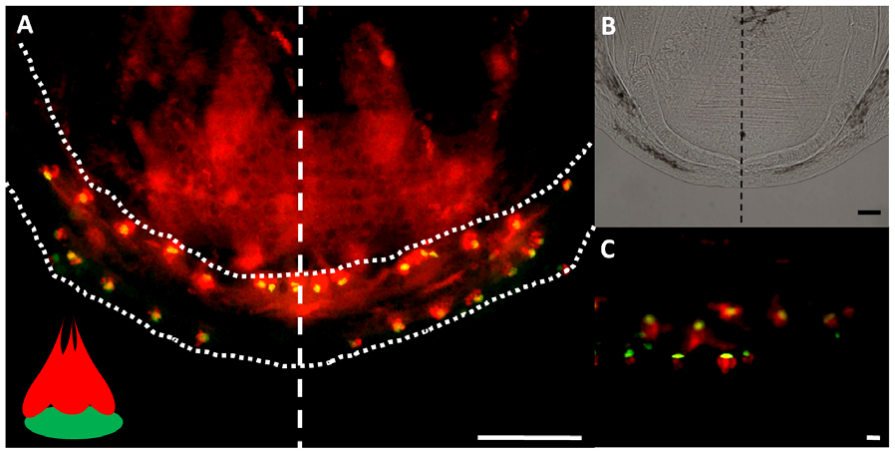
Immunohistochemical visualization of taste buds on the lower jaw at 21 dpf. (A) Flat mounted lower jaw (outlined in dotted line) showing the inner and outer rows of taste buds. A dashed line separates the control side from the surgery side. Each taste bud is visualized as one green basal cell grouped with one or more red receptor cells, as depicted in the schematic inset in (A). (B) Is a bright field image of the jaw in (A) at a lower magnification; (C) shows a higher magnification of the taste buds. All scale bars are 50 µm.

In order to determine whether lens ablation affects taste bud number, we performed ablations at 1 and 3 dpf and analyzed the number of taste buds on the surgery side of the mandible compared to the control side (Figures 5 and 6). Results indicate that when lens removal is performed at 1 dpf (the earliest time point at which the lens can be removed) and 3 dpf (after taste bud development has begun) the difference in taste bud count on the control versus surgery side of the lower jaw is not significant (paired t-test, p>0.05, n = 19 and n = 8 respectively). At 22 dpf, the surgery side does not have a two fold increase in the number of taste buds as expected in blind morphs [17]. Furthermore, the normal arrangement of taste buds in two rows is not altered in surgery specimens (100% of fish, n = 27) (Figures 5 and 6). These results indicate that lens removal does not significantly impact the number and arrangement of taste buds on the lips of the lower jaw after surgery (Figures 5 and 6).

No obvious alterations were observed in the pigmentation pattern on the surgery compared to the control sides (not shown), thus pigmentation is not influenced by lens removal.

### Lens ablation in the sighted tetra partially resembles the cavefish phenotype

**Figure 7:**
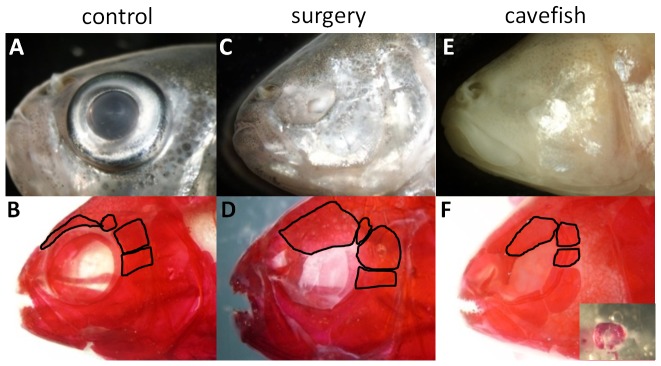
A comparison of normal surface fish, 1 dpf surgery fish and Tinaja cavefish. A, C, E are unstained. B, D, F are bone stained. In all stains SO4–6 and the supraorbital bone are outlined. (A–B) Adult control surface fish; (C–D) Show an adult surgery fish with surgery at 1 dpf; (E–F) Adult Tinaja cavefish; (F) The inset shows alizarin red stained scleral ossicles dissected from a regressed Tinaja cavefish eye.

We removed the lens in the surface morph embryos from 1 to 4 dpf to determine the developmental window during which time the developing eye can influence the development of the skull. We examined the effect of lens removal on the gustatory system and tooth development and are now in a position to determine the impact of the lens development on transitioning the surface fish morphology to a cavefish phenotype. Specifically, we wanted to determine whether lens removal in the surface fish would resemble the cavefish phenotype. We determined that there are some skeletal similarities shared between the surgery surface fish and the Tinaja cavefish. The supraorbital bone above the eye orbit is greatly expanded in the surgery fish, a similar expansion can be observed in the supraorbital bone of the cavefish (Figure 7). SO4–5 are expanded in the Tinaja cavefish in a similar manner to that observed in a 1 dpf surgery specimen. In addition, the orbit size is smaller in the surgery fish, similar to the cavefish. Scleral ossicles are present in most surgery fish and all Tinaja cavefish (Figure 7). When present, these ossicles are typically small and found arching around the small regressed eye. It is not possible to compare the Tinaja cavefish skull with that of the surgery surface fish with morphometrics as their skulls lack enough similarities to apply equivalent landmarks (e.g. an inconsistent number of circumorbital bones exist between the morphs). Our results do however show that some traits are shared between the surgery surface fish and the Tinaja cavefish that are not shared with a non-surgery surface fish.

## Discussion

The objective of this study was to build on previous work conducted by Jeffery and Yamamoto (2003) by and to determine the developmental window during which lens ablation affects adult skull morphology in the Mexican tetra. This type of long-term study is time consuming and therefore uncommon but provides valuable insight about the relationship between the development of the eye and the development of the surrounding skull.

### Lens ablation affects some craniofacial bones more than others

We removed the embryonic lens at 1 to 4 dpf then visualized the long term, permanent effects on the adult skeleton. In general teleost skeletons (trunk [21], [22], [23] and skull [24], [25], [26]) are extremely dynamic structures that display great plasticity. Only one other study has conducted lens ablations and these authors similarly observed changes in the circumorbital bones [11]. However, they minimally describe the effect of lens ablation at 1 dpf in a young adult skull. Here, we have expanded these time points to examine the developmental window that exerts the greatest effects on the skull. Our results show several expected and surprising results. First, we show that the bone most susceptible to change after lens removal is the supraorbital bone. This bone expands in all directions into a large plate covering much of the orbit. The second most affected bones were the SO4–6. Suborbital 4 and 5 are largely expanded, encroaching over the orbit and into the position normally occupied by SO6. Second, for the entire orbital complex, dorsal orbital bones are more affected than ventral orbital bones (Figures 2 and 4) suggesting that the source of the upstream events leading to this variation might be located dorsally (discussed below). Our results also indicate that there are different levels of plasticity in the bones surrounding the eye.

Most intriguing is that lens removal conducted within the first 4 dpf has tremendous effects despite orbital bones not ossifying until the fish reach approximately 3–4 months of age (equivalent to 1.6–2.2 cm standard length). As such the effects may be occurring early on in the skeletogenic condensations rather than during the ossification process.

There are a few possible explanations for the observed bone expansions into the eye orbit. In zebrafish (Cypriniformes: *Danio rerio*) the orbital bones are neural crest derived [27], however, their origin has not been determined in the Mexican tetra (Characiformes: *Astyanax mexicanus*) but is presumed to be similar. Cranial neural crest cells (CNCC) begin migration at 14 hours post fertilization [28] in zebrafish. Although the orbital bones are late forming bones, the cranial neural crest cells (CNCC) that give rise to them migrate to the area early in development, around the time of lens removal. We hypothesize that in order for the circumorbital bones to expand in size and shape an increase in cell number within the skeletogenic condensations may occur. This increase may arise through increased migration to the site or an increase in local cell proliferation. The presence of the eye has been shown to have an important function in the proper migration of cranial neural crest derived (CNCC) structures surrounding the eye [29], [30]. Langenberg and colleagues demonstrated using an eyeless zebrafish mutant, *chokh*/rx3, that the eye is necessary for the proper migration of the anterior neural crest cells into the dorsal part of the eye; when the eye is absent CNCC migration fails [29]. Skeletal defects in this mutant include malformations of the lower jaw and the neurocranium (i.e. anterior CNCC derived structures), suggesting that there is a failure in their development rather than a pleiotropic effect. Furthermore, when the lens vesicle is absent during early development these authors found that CNCC migration is arrested at the edge of the presumptive eye field. We hypothesize that lens removal may affect CNCC migration into the eye region, causing an increase in the migration of cells into the presumptive surrounding skeleton. In support of this, our data shows that the most affected bones are in the dorsal orbital region.

Alternatively lens removal may affect the bones surrounding the eye by a direct or indirect signalling or via a mechanical influence during later bone development. The lens may also function in a similar manner to the retina [30] by releasing paracrine factors [31]; these might inhibit orbital expansion under normal development. However, it is also important to note that mechanically the eye holds an important position within the head; when the eye is no longer present the bones of the skull are free to expand without constraint. In support of this alternative, the expansion of bones is always from the free edge of the bone (the non-sutured edge) and is always directed towards the space previously occupied by the eye. In addition, there are six extraocular muscles present in the zebrafish, which are thought to be conserved in all teleosts [32]. The extraocular muscles insert outside of the eye on the surrounding skeleton. Lens removal and subsequent eye regression may cause alterations in the development of these muscles which may in turn influence the development of the bones on which they insert. Alternatively expansion of these flat bones may be the result of increased appositional secretion of bone matrix. Further investigation is required to determine how the lens is influencing the surrounding skeleton; whether it is affecting the condensation through proliferation and/or migration, or whether it is affecting the growth of the ossified bone.

### Earlier lens ablation has a greater effect on the surrounding skull

Most intriguingly, our results indicate that the earlier lens ablation is conducted the greater the effect on the surrounding skeleton. That is, the supraorbital bone and suborbitals 4 to 6 (SO4–6) were expanded to a much greater extent when surgery was performed at 1 dpf compared to when surgery was performed at 4 dpf (Figure 2). This finding supports our hypothesis of a cellular basis for the observed effects rather than an underlying mechanical mechanism. As stated above, zebrafish neural crest cell migration begins early in development at approximately 14 hours post fertilization and continues in waves through the first few days of development [28]. The earlier the lens is removed the greater the time available to impact migration into the craniofacial region. When the lens is removed later, migration/proliferation may be nearly complete, thus allowing less time for lens removal to affect cellular dynamics.

The frontal and parietal bones are not affected by lens removal. We hypothesize that this might be due to a compensation effect by the bones encircling the eye. Bones such as the supraorbital and the suborbital bones (SO4–6) might be able to absorb the effects of lens removal by expanding such that the effects are not able to spread to the other more important areas of the skull such as the calvariae that protect the brain. Alternatively, the brain may provide protective signals to the overlying calvariae, thus maintaining their shape.

In addition to observing the influence of lens removal on the bone surrounding the eye, we also observed an effect on the bones within the eye. The scleral skeleton (present in the sclera of the eye) of the surface fish consists of two ossicles joined by cartilage. The sclera of the cavefish is thought to contain only cartilage, as a result of a failure to form scleral ossicles [11]. However, we determined that some cavefish populations do have ossified structures within the sclera in adults (Figure 7). Scleral ossicles form through endochondral ossification, with the anterior ossicle forming first [12]. The earlier we removed the lens, the greater the effect on the scleral ossicles; the greatest effect was always on the anterior ossicle which was either reduced or absent. This indicates, once again, that the earlier forming structures are affected to a greater degree by lens removal. The mechanism by which the lens is influencing the scleral skeleton is unknown and requires further investigation. Thompson et al. (2010) has suggested that scleral cartilage is induced by the pigmented retina, in chicken embryos; this process may be conserved in teleosts [33].

### The effects of lens ablation on taste bud development

The number of taste buds present in the oral region varies between the cavefish and the surface fish. Adult cavefish have more than twice the number of taste buds as surface fish [16]. In order to investigate the proposed links between the sensory modules [17], lens removal was conducted at 1 or 3 dpf in surface fish. Our results show that the absence of the lens does not affect the number or arrangement of taste buds. This analysis agrees with quantitative trait loci data which demonstrated that eye size is not significantly correlated to number of taste buds, despite cavefish having more taste buds [34]. However, Yamamoto et al. (2009) hypothesized that an increase in midline expression of *sonic hedgehog* (*Shh*) in the Mexican tetra has pleiotropic effects, which facilitates a trade-off between vision and taste. Similarities between our surgery fish and cavefish are discussed further below.

### The effects of lens ablation on the number of small mandibular teeth

The teeth of the Mexican tetra can be divided into three groups, based on differences in location within the mouth, timing of development and pattern of replacement [14]. In addition there are differences in the number of maxillary teeth between the two morphs. Surface fish typically have one multicuspid maxillary tooth while the Pachon cavefish have two or more maxillary teeth [11]. Only one study has investigated whether the lens influences tooth number [11]. When reciprocal lens transplants were performed between the cavefish and the surface fish the number of maxillary teeth were not affected. Based on this evidence, Yamamoto et al. (2003) concluded that maxillary teeth are not influenced by the transplanted cavefish lens. However, this study did not investigate the effects on tooth number outside of the maxillary region. At present there are also no studies that have investigated mandibular tooth number in cavefish. In our study, we show that lens removal does affect the number of small caudal teeth on the mandible; these teeth are spaced apart from one another. In addition, we determined that the large multicuspid mandibular teeth are unaffected by lens removal. Since more jaw space typically means more teeth [35] we analyzed the differences in the length of the jaw. We determined that the length of the mandible does not differ on the surgery side compared to the control side of the head and thus, jaw length cannot account for the observed changes in tooth number.

Due to the developmental differences between these types of teeth (age at development, and different replacement cycles) [11], [14]; it is likely that while the small caudal teeth appear to be influenced by the lens, the large multicuspid teeth are not susceptible to these influences. Interestingly, in the small eyed mouse mutant *Pax6^Sey^*/*Pax6^Sey^*, in which the lens fails to form; 80% of the mutants show an increase in anterior upper tooth number by 1 to 2 teeth [35], [36]. However, this is a pleiotropic effect. In mice, it has also been demonstrated that a single tooth can inhibit both the size and development of neighbouring teeth [37]. The isolated nature of the small teeth on the caudal portion of the mandible might enable them to be more plastic in number compared to the large clustered multicuspid teeth in the centre of the jaw.

Although little is known about the development of the small caudal teeth in the Mexican tetra, natural variation in tooth counts are reported within these teeth [14], again suggesting that these teeth are plastic. Small caudal teeth of the Mexican tetra are the only adult teeth that do not form within the jaw bones [14]. Their small size, the timing and manner of their development and their location close to the optic cup, might make these teeth more susceptible to influences from the developing eye than the central larger multicuspid teeth. Further investigation is required to understand this underlying mechanism.

### Does lens ablation in the surface fish resemble the cavefish phenotype?

We have shown that lens ablation conducted at 1–4 dpf in the surface fish affects the adult cranial skeleton and tooth number, but not taste bud number. We asked whether this phenotype resembles the cavefish phenotype. Overall, morphologically, the surgery surface fish have a cavefish-like craniofacial skeleton however many differences, such as the number of suborbital bones, exist between the skulls. The surgery phenotype may more closely resemble an intermediate morph (a F1 hybrid of a surface fish and a cavefish cross), however, no studies have documented the details of this phenotype. This comparison would be useful to better understand the transition from surface to cavefish phenotype.

Our study demonstrates that the regressing lens is one player in a much more complex system that led to the phenotypic changes in the derived cavefish. Increased midline expression of *Shh* in the cavefish leads to a decrease in *pax6* expression (a key eye development gene) [18]. Downstream effects of this increased expression include an up-regulation of the following genes *patched, vax1*, and *pax2A* in the head region and the eye [5], [38], [39], [40]. These genetic changes result in alterations in the lens, including a decrease in anti-apoptotic factors and an increase in pro-apoptotic factors [38]. According to Yamamoto et al. (2009) the changes in gene expression levels of *Shh* might be responsible for a decrease in cavefish eye size, increase in taste bud number and an increase in jaw size. Our results however, demonstrate that eye development does not affect taste bud number and jaw length in surface fish. It is important to note however, that this apparent difference in results might be explained by the level of investigation in each study (genetic level in Yamamoto study versus tissue level in our study). As a result we hypothesize that the observed phenotype of cavefish are unique characteristics of its genome that are not replicable in surface fish lacking an eye. The upstream signalling events that direct lens apoptosis are likely also responsible for the associated constructive and regressive changes observed in cavefish.

### Conclusion

We determined that lens removal conducted between 1 and 4 dpf influences the development of craniofacial bones surrounding the eye; most dramatically affecting the supraorbital bone and suborbital bones 4 through 6, while the calvariae and the jaw are unaltered. Interestingly, lens removal had the greatest effect on the skull the earlier it was conducted. The affected bones are more plastic in their development than others in the skull and are more influenced by the eye due to their closer association with it. Scleral ossicles were either reduced or absent as a result of lens removal with the earlier developing anterior ossicle most affected. In addition, we determined that the lens has the ability to influence the number of small caudal teeth on the lower jaw. Finally, we determined that the lens does not influence the development of taste buds (as seen in cavefish) or the length of the lower jaw. We conclude that the cavefish phenotype, which arises due to complex signaling leading to lens apoptosis, cannot be replicated by ablating the lens in surface fish. With this long term study we have demonstrated that the lens, a soft tissue in the head, has the capacity to influence the development of particular bones that develop several months later. Whether the lens influences the skull through a direct or indirect, mechanical or molecular manner has yet to be determined. Overall, this research raises many questions regarding the role of the eye in directing development of the vertebrate head.

## Supporting Information

Table S1 **The 42 landmark locations used for the lateral view of the head.**
(DOCX)

Table S2 **The eleven landmark locations used on the dorsal view of the skull.**
(DOCX)

Table S3 **The eleven landmark locations used on the ventral view of the lower jaw.**
(DOCX)

Table S4 **Landmark groups identified based on their similar response post surgery.**
(DOCX)
