# Supplementary material for: Early Lens Ablation Causes Dramatic Long-Term Effects on the Shape of Bones in the Craniofacial Skeleton of Astyanax mexicanus
Source: PLoS One. 2012 Nov 30;7(11):e50308. doi: 10.1371/journal.pone.0050308 (PMC3511446; doi:10.1371/journal.pone.0050308)
Supplement: Table S1 — The 42 landmark locations used for the lateral view of the head. (DOCX) [file pone.0050308.s001.docx]

| **Landmark Number** | **Location of landmark** |
| --- | --- |
| Landmark 1 | dorsal edge of the mandibular symphysis |
| Landmark 2 | ventral edge of the mandibular symphysis |
| Landmark 3 | anterior corner of the mandible |
| Landmark 4 | midline posterior corner of the coronoid process |
| Landmark 5 | center of posterior edge of the coronoid process |
| Landmark 6 | lateral posterior corner of the coronoid process |
| Landmark 7 | midline anterior corner of the coronoid process |
| Landmark 8 | midline anterior corner of the coronoid process |
| Landmark 9 | anterior most point of the preopercle |
| Landmark 10 | anterior most point of the quadrate |
| Landmark 11 | anterior most point of the interopercle |
