# Supplementary material for: Early Lens Ablation Causes Dramatic Long-Term Effects on the Shape of Bones in the Craniofacial Skeleton of Astyanax mexicanus
Source: PLoS One. 2012 Nov 30;7(11):e50308. doi: 10.1371/journal.pone.0050308 (PMC3511446; doi:10.1371/journal.pone.0050308)
Supplement: Table S2 — The eleven landmark locations used on the dorsal view of the skull. (DOCX) [file pone.0050308.s002.docx]

| **Landmark Number** | **Location of landmark** |
| --- | --- |
| Landmark 1 | anterior tip of the supraethmoid |
| Landmark 2 | center of posterior edge of the supraethmoid |
| Landmark 3 | lateral posterior corner of the supraethmoid |
| Landmark 4 | midline anterior corner of the frontal bone |
| Landmark 5 | center anterior edge of the supraorbital |
| Landmark 6 | center posterior edge of the supraorbital |
| Landmark 7 | lateral posterior corner of the frontal bone |
| Landmark 8 | midline posterior corner of the frontal bone |
| Landmark 9 | lateral posterior corner of the parietal bone |
| Landmark 10 | center of the posterior edge of the parietal bone |
| Landmark 11 | midline posterior corner of the parietal bone |
