# Supplementary material for: Early Lens Ablation Causes Dramatic Long-Term Effects on the Shape of Bones in the Craniofacial Skeleton of Astyanax mexicanus
Source: PLoS One. 2012 Nov 30;7(11):e50308. doi: 10.1371/journal.pone.0050308 (PMC3511446; doi:10.1371/journal.pone.0050308)
Supplement: Table S3 — The eleven landmark locations used on the ventral view of the lower jaw. (DOCX) [file pone.0050308.s003.docx]

| **Landmark Number** | **Location of landmark** |
| --- | --- |
| Landmark 1 | anterodorsal corner of the supraorbital bone |
| Landmark 2 | anteroventral corner of the supraorbital bone |
| Landmark 3 | posterodorsal corner of the supraorbital bone |
| Landmark 4 | posterodorsal corner of the supraorbital bone |
| Landmark 5 | anterodorsal corner of suborbital 5 |
| Landmark 6 | posterodorsal corner of suborbital 5 |
| Landmark 7 | anteroventral corner of suborbital 5 |
| Landmark 8 | posterodorsal corner of suborbital 5 |
| Landmark 9 | anterodorsal corner at suborbital 4 |
| Landmark 10 | posterodorsal corner of suborbital 4 |
| Landmark 11 | anteroventral corner of suborbital 4 |
| Landmark 12 | posterodorsal corner of suborbital 4 |
| Landmark 13 | anterodorsal corner of suborbital 3 |
| Landmark 14 | posterodorsal corner of suborbital 3 |
| Landmark 15 | the notch on the ventral edge of suborbital 3 |
| Landmark 16 | anteroventral corner of suborbital 3 |
| Landmark 17 | anterodorsal corner of suborbital 3 |
| Landmark 18 | posterodorsal corner of suborbital 2 |
| Landmark 19 | posterodorsal corner of suborbital 2 |
| Landmark 20 | anteroventral corner of suborbital 2 |
| Landmark 21 | anterodorsal corner of suborbital 2 |
| Landmark 22 | posterodorsal corner of suborbital 1 |
| Landmark 23 | posterodorsal corner of suborbital 1 |
| Landmark 24 | anterodorsal corner of suborbital 1 |
| Landmark 25 | anteroventral corner of suborbital 1 |
| Landmark 26 | the ventral tip of the antorbital bone |
| Landmark 27 | the dorsal tip of the antorbital bone |
| Landmark 28 | posterodorsal corner of the nasal bone |
| Landmark 29 | anteroventral corner of the nasal bone |
| Landmark 30 | the center of dorsal edge of the nasal bone |
| Landmark 31 | ventral posterior corner of the frontal bone |
| Landmark 32 | the posterior midline corner of the frontal bone |
| Landmark 33 | anterior edge of the frontal bone |
| Landmark 34 | dorsal edge of the frontal bone where a drastic downward slope begins |
| Landmark 35 | top most point of the maxilla |
| Landmark 36 | ventral most point at the bottom of the maxilla |
| Landmark 37 | center of the ventral edge of the lateral ethmoid |
| Landmark 38 | center of the dorsal edge of the lateral ethmoid |
| Landmark 39 | anterodorsal corner of suborbital 6 |
| Landmark 40 | anteroventral corner of suborbital 6 |
| Landmark 41 | posterodorsal corner of suborbital 6 |
| Landmark 42 | posterodorsal corner of suborbital 6 |
