# Supplementary material for: Early Lens Ablation Causes Dramatic Long-Term Effects on the Shape of Bones in the Craniofacial Skeleton of Astyanax mexicanus
Source: PLoS One. 2012 Nov 30;7(11):e50308. doi: 10.1371/journal.pone.0050308 (PMC3511446; doi:10.1371/journal.pone.0050308)
Supplement: Table S4 — Landmark groups identified based on their similar response post surgery. (DOCX) [file pone.0050308.s004.docx]

| **Group** | **Landmarks** | **Bones** |
| --- | --- | --- |
| **1** | 1-10 and 39-42 | Supraorbital, suborbital 6, suborbital 5 and the dorsal edge of suborbital 4 |
| **2** | 11-14 | Ventral end of suborbital 4 and the dorsal edge of suborbital 3 |
| **3** | 15-23 and 36-37 | Ventral part of suborbital 3, suborbital 2, posterior edge of suborbital 1 and the lateral ethmoid |
| **4** | 24-30 and 33-36 | Anterior edge of suborbital 1, antorbital bone, nasal bone, frontal bone, and the maxilla |
